# Green Tea and Benign Gynecologic Disorders: A New Trick for An Old Beverage?

**DOI:** 10.3390/nu15061439

**Published:** 2023-03-16

**Authors:** Dana Hazimeh, Gaelle Massoud, Maclaine Parish, Bhuchitra Singh, James Segars, Md Soriful Islam

**Affiliations:** Department of Gynecology and Obstetrics, Division of Reproductive Sciences & Women’s Health Research, Johns Hopkins Medicine, Baltimore, MD 21205, USA

**Keywords:** green tea, EGCG, fibroids, adenomyosis, dysmenorrhea, endometriosis, infertility, polycystic ovary syndrome (PCOS), menopause

## Abstract

Green tea is harvested from the tea plant *Camellia sinensis* and is one of the most widely consumed beverages worldwide. It is richer in antioxidants than other forms of tea and has a uniquely high content of polyphenolic compounds known as catechins. Epigallocatechin-3-gallate (EGCG), the major green tea catechin, has been studied for its potential therapeutic role in many disease contexts, including pathologies of the female reproductive system. As both a prooxidant and antioxidant, EGCG can modulate many cellular pathways important to disease pathogenesis and thus has clinical benefits. This review provides a synopsis of the current knowledge on the beneficial effects of green tea in benign gynecological disorders. Green tea alleviates symptom severity in uterine fibroids and improves endometriosis through anti-fibrotic, anti-angiogenic, and pro-apoptotic mechanisms. Additionally, it can reduce uterine contractility and improve the generalized hyperalgesia associated with dysmenorrhea and adenomyosis. Although its role in infertility is controversial, EGCG can be used as a symptomatic treatment for menopause, where it decreases weight gain and osteoporosis, as well as for polycystic ovary syndrome (PCOS).

## 1. Introduction

Tea may be the most consumed beverage worldwide, with green tea (*Camellia sinensis*) being the most popular tea variety [1]. The major active compound in green tea is epigallocatechin gallate, commonly known as EGCG. EGCG has been studied as a cancer therapeutic and exhibits chemopreventive effects in many cancer types, including breast, colon, prostate, liver, ovary, uterus, and kidney cancer [2,3,4,5,6]. EGCG is mechanistically complex, as it can act as both an antioxidant and a prooxidant based on the unique cellular conditions and dosage [7]. Based on these actions, EGCG is clinically promising because it targets a variety of pathways, including apoptosis, lipid peroxidation, cholesterol sequestration, and free radical scavenging [8,9,10]. There are many diseases for which EGCG might prove to be of clinical benefit. This review highlights the chemical and biological properties of EGCG, as well as its mechanisms of action, and serves as an introduction to understanding the use of EGCG in the treatment of benign women’s reproductive disorders.

Benign reproductive disorders, including endometriosis, fibroids, adenomyosis, dysmenorrhea, and polycystic ovary syndrome (PCOS), affect millions of women worldwide. These conditions are very common. The prevalence of endometriosis ranges from 10–15% [11]. Fibroids alone can impact upwards of 65% of women at some point in their lives, and PCOS affects 20% of women [12,13]. Many of these conditions result in a significant adverse impact on the quality of life for affected women because of symptoms such as heavy menstrual bleeding, iron deficiency, and pelvic pain [14]. Furthermore, these conditions may cause secondary health effects, such as infertility [14]. Currently, low-risk, non-hormonal primary and secondary preventive treatment options for these conditions are limited. This review explores how EGCG may provide health benefits and reduce symptoms associated with common gynecological conditions.

## 2. Methods

This article provides a comprehensive scoping review of the available literature discussing the role of green tea in various benign gynecological disorders, including data from in vivo, in vitro, and clinical studies. A literature search was conducted until January 2023 using electronic databases, including PubMed of the National Library of Medicine, Google Scholar, and relevant clinical trials addressing green tea and benign gynecological disorders. The keywords “green tea”, “EGCG”, “chemical composition”, “bioavailability”, ”metabolism”, “pharmacokinetics”, “pharmacodynamics”, “EGCG mechanisms”, “antioxidant”, “prooxidant”, “dysmenorrhea”, “adenomyosis”, “endometriosis”, “uterine leiomyoma”, “uterine fibroid”, “polycystic ovary syndrome”, “menopause”, “osteoporosis”, “weight loss”, “infertility” were used. Only papers published as full-length articles in English were considered.

## 3. Green Tea

### 3.1. Green Tea: Overview

**Figure 1:**
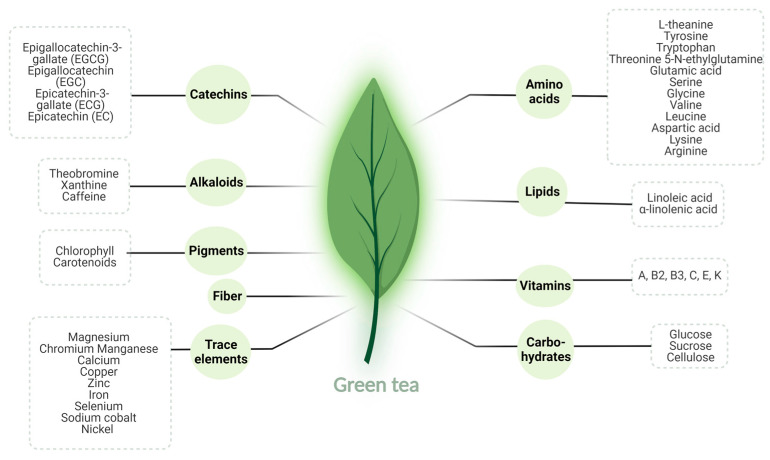
Chemical composition of green tea.

Green tea is one of the most consumed beverages in the world, accounting for about 20% of total tea consumption worldwide [15,16]. Its various health benefits have been widely determined, including its important therapeutic potential in the pathologies of the female reproductive system [6,16,17]. Green tea contains a substantial variety of nutrients, including carbohydrates, lipids, amino acids (notably L-theanine), alkaloids (caffeine, xanthine, and theobromine), vitamins (A, B2, B3, C, E, and K), and pigments like chlorophyll and carotenoids. It is also a rich source of trace elements such as calcium, zinc, iron, copper, chromium, magnesium, and manganese [18,19] (Table 1). Polyphenolic compounds known as catechins constitute 30% of the dry green tea leaf weight, and these mainly include epigallocatechin-3-gallate (EGCG), epicatechin-3-gallate (ECG), epigallocatechin (EGC), and epicatechin (EC) (Figure 1). Considerable evidence suggests that EGCG is the most active catechin derivative and the main contributor to green tea’s favorable research findings [2,20,21].

Catechins exhibit antioxidant properties, as demonstrated by many previous studies. For instance, catechins were shown to have a higher antioxidant capacity than vitamins C and E. In fact, the antioxidant power of one cup of green tea was estimated to be equivalent to 100–200 mg of pure ascorbic acid [22,23,24,25,26]. This high level of antioxidant property is due to the fact that green tea is not fermented during its manufacturing, and therefore it retains high catechin levels in its composition. During the fermentation process of other types of tea (black or oolong tea, for example), catechins undergo an auto-oxidation reaction catalyzed by the enzyme polyphenol oxidase, to form polymers known as theaflavins and thearubigins. In contrast, catechins in unfermented green tea are not oxidized, and their powerful chemical properties remain intact [18,19,27,28]. Green tea therefore has a significantly higher catechin content and is thought to exert a more potent antioxidant activity compared to other teas [2,19,20,27,28,29,30].

### 3.2. Green Tea: Pharmacokinetics and Bioavailability

After oral ingestion of green tea, its structural stability improves in the stomach due to the acidic pH [31]. The catechins are mainly absorbed in the intestine, specifically in the jejunum and ileum, by the monocarboxylate transporter (MCT). Catechins can be absorbed by passive diffusion or active carriers, including MCT and multidrug resistance-associated protein 2 (MRP2) [32]. MRP2, as well as P-glycoprotein (P-gp), are transporters that act to efflux EGCG from the apical membrane of the intestinal cells. This affects the absorption of several drugs that interact with MRP1, MRP2, and P-gP when consumed with green tea [33,34]. For instance, the bioavailability of the chemotherapeutic agent irinotecan is increased when combined with EGCG due to the inhibition of the P-gP transporter by EGCG [35]. Green tea catechins also inhibit other drug transporters, such as the organic anion transporting polypeptides (OATPs). Both atorvastatin and nadolol absorption is decreased when consumed with green tea due to the inhibition of the OATPs [36,37]. Although green tea can alter the bioavailability of certain drugs, its own absorption can also be affected by the presence of food. It has been observed that fish oil, vitamin C, and carbohydrates increase the absorption of EGCG [38,39]. The bioavailability of EGCG was increased when administered with ascorbic acid and sucrose, compared to green tea alone, in rats (Area Under the Curve (AUC) = 181.8 pmol·h/L vs. 61 pmol·h/L) [39]. Intestinal absorption of total catechins was enhanced 6 and 11 times when taken with xylitol/citric acid and xylitol/vitamin C, respectively [40]. Similarly, the bioavailability of flavanols was increased by 140% when ingested with carbohydrates, especially sugar and bread [41]. Optimal bioavailability is achieved in a fasting state. The absorption of EGCG increased 2.7 and 3.9 times when ingested while fasting compared to when taken with either a light breakfast or strawberry sorbet, respectively [42]. Peak plasma concentration was reached after 90 min of oral ingestion [43]. In the plasma, EGCG is mainly present in the sulfate form (58–72%), followed by the free form (3–13%), and then the glucuronide form (8–19%) [44]. EGCG then becomes undetectable in plasma after 24 h [45]. EGCG diffuses well in different body tissues and compartments. It can reach the brain by crossing the blood-brain barrier [46,47]. Catechins were also found in prostatic tissue [48]. In rodents, the volume of distribution of EGCG was approximately 0.3–0.7 L/kg [49]. EGCG was also distributed in different organs, similarly to humans, including the esophagus, large intestine, and bladder, but it was not detected in the lungs, spleen, or liver [50].

Catechins mainly undergo phase II metabolic reactions, including glucuronidation, sulfation, and methylation, which occur in the liver and intestines after oral ingestion. Sulfation and glucuronidation increase the polarity of catechins to increase their urinary excretion [44,51]. Methylation of EGCG yielded predominantly di-methoxyl-EGCG (di-OMe-EGCG) via methylation by the catechol-*O*-methyltransferase (COMT) [52]. A substantial amount of EGCG that is excreted through bile is also broken down by the colonic bacteria to ring-fission metabolites [53,54]. The metabolites are reabsorbed in the plasma and eliminated in the urine. Catechin catabolites were phenylvalerolactones and phenylvaleric acid, including 5-(3′,4′,5′-trihydroxyphenyl)-γ-valerolactone (M4) and 5-(3′,4′-dihydroxyphenyl)-γ-valerolactone (M6) [45]. The compounds were further metabolized to aromatic and phenolic acids to be reabsorbed in the circulation and eliminated in the urine as pyrogallol-2-O-sulfate, pyrogallol-2-O-glucuronide, valerolactone-3′-O-sulfate, and vanilloylglycine after being conjugated [55].

The pharmacokinetic parameters of EGCG have been widely studied, and data suggest that EGCG has a half-life (*t1/2*) of 5 ± 2 h and reaches its peak plasma concentration at approximately 2 h after ingestion [45,56,57,58,59]. Green tea EGCG has low bioavailability [60]. Several groups have used synthetic EGCG derivatives, also known as EGCG prodrugs, generated by acetylation of the reactive hydroxyl groups. This helped overcome EGCG’s low bioavailability and improved its biological potency [61]. For instance, in murine endometrial implants and human leiomyoma cells, pro-EGCG analogs exhibited greater anti-oxidative and anti-angiogenic capacity than EGCG [62,63]. Their anti-tumor effects were also stronger than those observed with EGCG, as demonstrated in experimental cancer mouse models and different cancer cell lines [64,65,66,67,68]. Together, these findings provide insight into the use of EGCG prodrugs for their more favorable chemical and therapeutic properties.

### 3.3. Green Tea: Mechanisms of Action

*EGCG interacting with proteins*: EGCG has been reported to interact with transmembrane receptors or proteins involved in disease progression. For example, EGCG can bind prolyl cic/trans isomerase, 67 kDa laminin receptors, and glucose-regulated protein 78, proteins known to be important in cancer metastasis and cell invasion pathways [69,70,71]. Moreover, EGCG was found to bind transforming growth factor-beta (TGF-β) type II receptor (TGFR-II) and inhibit the downstream activity of TGF-β in cellular differentiation and migration pathways [72]. EGCG has also been found to directly interact with many components of the extracellular matrix (ECM), including fibrinogen, fibronectin, and histidine-rich glycoprotein [73]. Similarly, EGCG can interact with vimentin, an intermediate filament [74]. Several metalloproteinases (MMPs) are also known to have binding interactions with EGCG, including MMP-2, MMP-9, MMP-12, and MMP-9, although EGCG binds MMP-9 and MMP-12 with the highest affinity [75,76]. EGCG was found to occupy the ATP binding site of P-gp, the efflux transporter pump mentioned previously, and inhibit its nucleotide binding domain 2 [77]. EGCG also interacts with key proteins involved in apoptosis. It increases the pro-apoptotic activity of the death receptor protein Fas [78,79] and binds antiapoptotic proteins such as B-cell lymphoma-extra large and B-cell lymphoma-2 [78,80].

**Figure 2:**
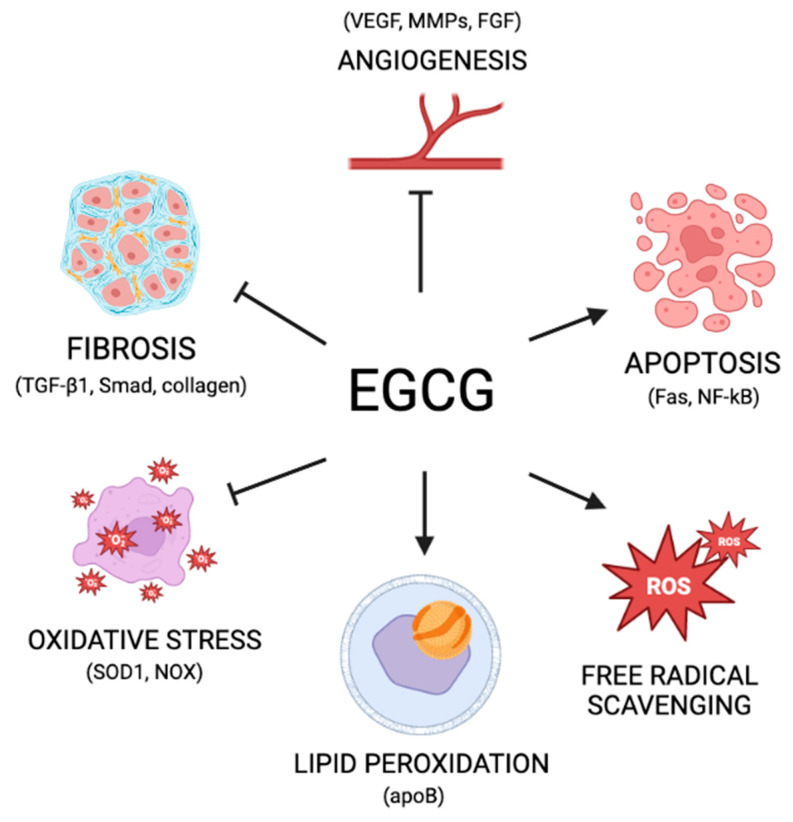
Mechanisms of action of EGCG.

*EGCG as an antioxidant:* Green tea and its major compound, EGCG, have been widely studied for their therapeutic properties. The proposed mechanisms by which EGCG modulates cellular activities and environments are complicated, as it can function as both a prooxidant and an antioxidant (Figure 2).

As an antioxidant, EGCG can play many roles. Much of its antioxidation capability is attributed to the unique catechin structure of EGCG. All green tea catechins are composed of a saturated heterocyclic ring important for electron delocalization in the scavenging of free radicals [79]. These phenolic structural motifs allow catechins to act as hydrogen donors to directly interact with free radicals [81]. Unlike other green tea catechins, EGCG also has a gallate group, allowing it to act as a more potent antioxidant by increasing its ability to act as an electron donor [81]. Apart from directly scavenging free radicals, EGCG can also promote antioxidant actions directly through chelating transition metals [82]. Transition metals are often involved in the autooxidation reactions that generate free radicals, so by inhibiting the progression of such reactions, EGCG can further limit free radical accumulation.

EGCG can also facilitate antioxidant effects indirectly through the regulation of other components important in antioxidant mechanisms. For example, EGCG has been found to drive the expression of superoxidase dismutase, an enzyme that can facilitate the breakdown of superoxide, the primary free oxygen radical, into molecular oxygen and hydrogen peroxide [7]. Similarly, EGCG was found to reduce several compounds implicated in prooxidant activity. For example, EGCG can dampen the expression of nicotinamide adenine dinucleotide phosphate oxidase (NOX), an enzyme responsible for catalyzing the transfer of electrons to oxygen molecules in the generation of reactive oxygen species (ROS) [83].

These antioxidant functions have many downstream effects, allowing EGCG to effectively inhibit the hazardous reactions that can occur if free radical concentration is dysregulated. For example, EGCG has been implicated as an inhibitor of lipid peroxidation, a downstream consequence of high free radical concentrations. Dangerous reactive aldehydes are generated as by-products when free radicals oxidize lipids [84]. Low-density lipoprotein (LDL) cholesterol is a lipid compound susceptible to oxidation when allowed to build up on the walls of arteries to form atherosclerotic plaques. EGCG has been shown to inhibit the oxidation of LDL cholesterol in these contexts through its function as an antioxidant to decrease free radical availability [85]. In addition to the indirect effect of free radical sequestration on the oxidation of LDL cholesterol, it has been shown that EGCG can physically interact with LDL particles to physically prevent their oxidation [9]. This suggests the effect of EGCG on the regulation of LDL cholesterol is more complex, encompassing more than merely downstream consequences of antioxidant activity. Apart from its impact as an antioxidant, EGCG can function to sequester LDL cholesterol from the bloodstream to prevent atherosclerotic plaques from forming. EGCG can inhibit apolipoprotein B (apoB) secretion, an important chaperone protein required for LDL cholesterol to remain in circulation [86]. Through this, LDL binds LDL receptors (LDLRs) on the plasma membrane to be stored as lipid droplets in the cytoplasm, thus decreasing the concentration of circulating LDL [8].

*EGCG as a prooxidant:* EGCG can facilitate prooxidant effects through many mechanisms. These effects are less commonly studied and discussed but are important to consider. EGCG can act through both direct and indirect mechanisms as a prooxidant compound. Directly, EGCG can undergo its own autooxidation reactions that produce superoxide free radicals and other reactive oxygen species [87]. Interestingly, this reaction is driven by the same structural properties that make EGCG such a potent antioxidant. The trihydroxy groups on the phenol rings of EGCG make it an excellent electron donor, allowing EGCG to generate superoxide by providing electrons to molecular oxygen particles as a result of its auto-oxidation [88]. Through similar principles, EGCG can promote Fenton reactions, reducing FeIII to FeII through electron donation [82]. Here, ROS are produced as a byproduct [82].

Apoptosis is the main activated pathway, which is attributed to the role of EGCG as a prooxidant. EGCG has been found to drive internal (mitochondrial-driven) and external (ligand-receptor-mediated) apoptotic mechanisms. In internally driven apoptosis, B-cell lymphoma-2 (Bcl2) associated X protein (BAX) activity drives the mitochondrial release of cytochrome C to stimulate the caspase cascade that triggers apoptosis [89]. In a liver cancer model, EGCG treatment led to a downregulation of nuclear factor κ-B (NF-κB), a cell proliferation signal. As a result, Bcl-2 dissociates from BAX, allowing BAX to drive a decrease in mitochondrial membrane potential to stimulate cytochrome C release [4]. Importantly, this effect was restricted to cancer cells, allowing healthy cells to remain functioning normally, although the mechanism driving this selection remains unclear [4].

In ligand-receptor-mediated apoptotic pathways, similar impacts on apoptosis were facilitated through regulation of the Fas death receptor. In this mechanism, ligand binding to the Fas cell membrane receptor directly stimulates the binding of the initiator caspase, caspase-8 [90]. In an adrenal cancer model, caspase-8 activity was upregulated following EGCG treatment, suggesting EGCG may be activating the activity of Fas [91]. Similar effects have been observed in many models of human cancer, including leukemia, head and neck squamous, pancreatic, and liver cancer [92,93,94,95].

The antioxidant and prooxidant mechanisms outlined above (Figure 2) are important to understand when attempting to decipher the impact EGCG may have on pathways mediating disease states. The balance between antioxidant and prooxidant effects determines the therapeutic impact of EGCG. This balance has been largely attributed to dosage. At higher doses (1–100 μM), EGCG behaves as a prooxidant, while lower doses (0.1–0.01 μM) favor its antioxidant effects [87]. This understanding is important when considering clinical applications. For example, at low doses, EGCG could be used as a cancer prevention strategy, as it promotes cell survival and prevents DNA damage by limiting oxidative stress as an antioxidant. On the other hand, at high doses, EGCG could be used as a cancer therapeutic, targeting later-stage cancer cells to drive apoptosis and induce cellular damage to fight tumor progression. However, the functions are not so easily distinguished from one another, as EGCG commonly inflicts both prooxidant and antioxidant effects simultaneously [96].

*EGCG: anti-angiogenic effects:* The formation of new blood vessels through angiogenesis is a process essential to cancer invasion and metastasis. The bloodstream provides nutrients to the tumor, allowing it to thrive and grow. This process is especially important in women’s reproductive health because reproductive tissues are highly vascularized and experience cyclic intervals of growth and revascularization [97]. In both cases, this process is largely driven by vascular endothelial growth factors (VEGFs) and fibroblast growth factors (FGFs) [97]. It has been shown both in vitro and in vivo that EGCG can significantly inhibit angiogenesis [98,99]. This effect is probably due to the compounding impact of several levels of EGCG intervention, including direct effects on proangiogenic factors (Figure 2). Primarily, EGCG has been observed to inhibit VEGF and FGF, directly inhibiting angiogenesis [100,101,102]. In addition, EGCG is known to have direct inhibitory binding interactions with many matrix metalloproteinases (MMP), proteins that promote ECM remodeling and drive the release of pro-angiogenic compounds such as VEGF [75]. In this way, EGCG can also inhibit angiogenesis through the inhibition of MMPs [103].

*EGCG: antifibrotic effects*: Fibrosis, broadly defined as the buildup of ECM components as a result of improper connective tissue repair, can occur in a wide range of tissue and organ types [104]. TGF-β1 is widely characterized as a mediator of fibrosis, largely through activating small mother against decapentaplegic (Smad) signaling to drive scarring and the deposition of collagen [105]. These processes are crucial in understanding the progression of fibrosis in many organ types. EGCG has been most prominently characterized as a potential fibrotic inhibitor in idiopathic pulmonary fibrosis (IPF), although it has also been studied in liver, cardiac, uterine, dermal, and renal fibrosis [106,107,108,109,110]. One of the important mechanisms thought to mediate this effect is EGCG driven inhibition of TGF-β1 in fibroblasts [72]. As an anti-fibrotic, EGCG has been shown to downregulate components of the Smad signaling pathway to decrease proliferation and overproduction of collagen [111] (Figure 2).

## 4. Role of Green Tea against Benign Gynecological Disorders

### 4.1. Green Tea and Dysmenorrhea

Dysmenorrhea is a pelvic pain condition that occurs prior to or during menstruation and affects 45% to 93% of women of reproductive age [112]. This condition can be further classified as either primary or secondary dysmenorrhea. While secondary dysmenorrhea refers to pelvic pain resulting from a gynecological disorder, patients with primary dysmenorrhea experience painful menstruation yet lack evidence of pelvic pathology [113]. Secondary dysmenorrhea can be the result of conditions such as fibroids, endometriosis, or adenomyosis. The role of EGCG in the specific treatment of these gynecologic conditions will be discussed separately.

From a mechanistic standpoint, primary dysmenorrhea is associated with overproduction of prostaglandins in the endometrium [114]. In prostaglandin biosynthesis, arachidonic acid is converted to prostaglandin through the cyclooxygenase (COX) pathway [115]. During menstruation, the COX pathway is activated by a natural drop in progesterone, allowing for phase-specific expression of prostaglandins [116]. Since prostaglandins are hormone-like lipids that are typically responsible for responding to areas of infection or damage, an overproduction can change the uterine environment to induce hypoxic conditions, uterine muscle ischemia, and increased nerve sensitivity [117]. Overall, this results in uterine hypercontractility, which ultimately leads to pain.

**Figure 3:**
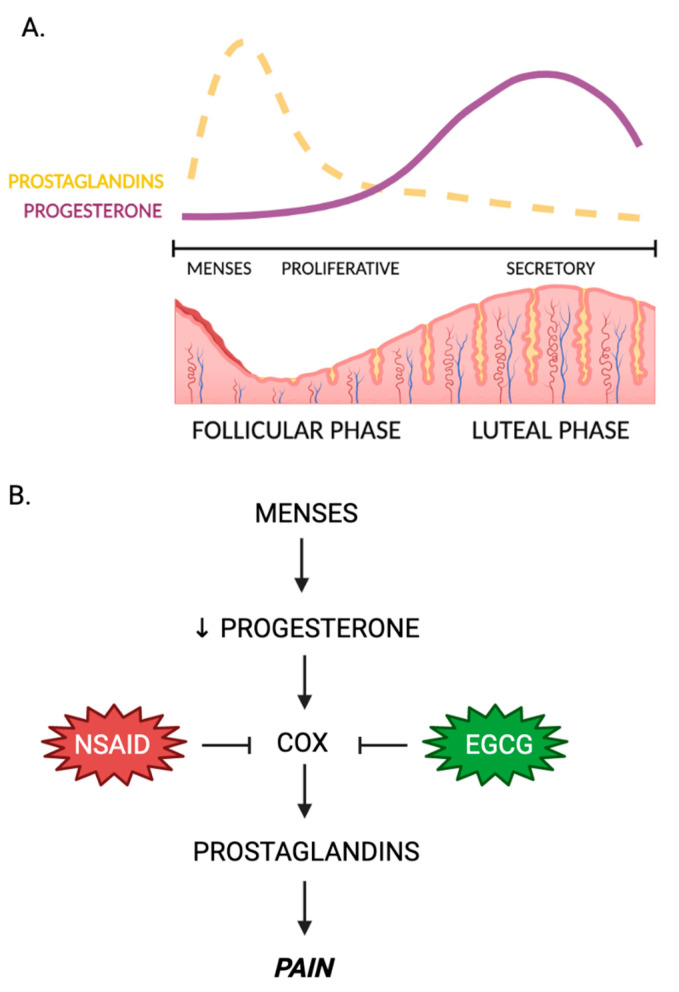
Possible actions of EGCG in dysmenorrhea. (**A**) Progesterone and prostaglandin concentrations are inversely related. During menses, a fall in progesterone activity stimulates the COX pathway to begin synthesizing prostaglandins. (**B**) A mechanistic scheme of the effects of NSAIDs and EGCG on the regulation of prostaglandin concentrations. Here, prostaglandin concentration is directly correlated with pain. ↓ = downregulation.

Currently, over-the-counter contraceptive pills (OCPs) and non-steroidal anti-inflammatory drugs (NSAIDs) are used to treat primary dysmenorrhea [118]. However, there is a need to expand therapeutic options for women with primary dysmenorrhea, as the current treatments present both physiological and efficacy concerns [118,119]. Studies showed that EGCG can limit the biosynthesis of prostaglandins, such as prostaglandin E2 (PGE2) [5]. Currently, NSAIDs target the COX pathway, decreasing prostaglandin accumulation to result in a decrease in pain intensity. EGCG provides a natural alternative to NSAIDs that may function through similar mechanisms. In a study of chronic inflammation and its link to cancer, it was found that EGCG could limit PGE2 synthesis in both cell-free and human whole blood assays, likely through inhibition of microsomal prostaglandin E synthase-1 (mPGES-1), an enzyme important in catalyzing the COX pathway [120]. This conclusion was further validated in a later study in human monocytes [121]. Together, these preliminary data suggest EGCG may modulate prostaglandin synthesis via the COX pathway and thus may provide relief to women with dysmenorrhea (Figure 3).

In a cross-sectional study conducted in Shanghai, China, from August 2013 to April 2015, 1183 women of childbearing age were monitored for self-reported menstrual and pelvic pain [119]. They were asked to grade their pain on a set scale, selecting from mild, moderate, and severe. These women were also asked lifestyle questions, such as about their tea-drinking behavior. Overall, tea drinking was associated with a lower prevalence of dysmenorrhea, with green tea exhibiting the strongest reduction when compared to other tea varieties. This reduction was stronger in women reporting moderate to severe pelvic pain. While this study did not assess the exact amount of green tea intake, the authors [119] suggest green tea and its major active compound, EGCG, act through modulation of the COX pathway to reduce menstrual pain in study participants, although more studies are needed to validate these findings.

### 4.2. Green Tea and Infertility

Infertility, or the inability to get pregnant after one year of unprotected intercourse, is a common problem. In the United States, the Centers for Disease Control report that approximately 1 in 5 (19%) heterosexual women aged 15 to 49 are infertile [122]. Many factors come into play, including structural anomalies and environmental factors; however, age remains the most important one [122].

Oxidative stress, which is characterized by a state of imbalance between pro-oxidant reactive nitrogen, oxygen species, and antioxidant molecules, is a major contributing factor to infertility. Excess ROS affect oocyte maturation. Interestingly, meiosis is induced by a rise in ROS, including hydrogen peroxide (H_2_O_2_), and is inhibited by an increase in antioxidants [123,124]. In contrast, the progression of meiosis II is stimulated by a rise in antioxidants, which highlights the importance of a balanced oxidative state in the ovary. Green tea, known for its antioxidative properties, reduced ROS production, thus improving the quality of female gametes [125]. EGCG also reduced the pro-apoptotic protein expression of Bax and caspase-3 and increased the anti-apoptotic protein expression of Bcl-2 in mature oocytes [125].

Green tea and its major bioactive component, EGCG, improved the outcomes of in vitro fertilization (IVF) in several species. In fact, several studies showed that treatment with low doses of green tea polyphenols (10, 15 µM) improved the maturation of bovine and porcine oocytes and the proportion of blastocysts at in vitro insemination [125,126]. This improvement might be attributed to the increase in intracellular glutathione concentrations [126]. Similarly, improvements in the formation rates of morula and blastocysts as well as the maturation of sheep oocytes were noted when treated with a low dose of green tea extract (0.3 mg/mL) [127]. Treatment with high doses of EGCG did not show any benefits in IVF outcomes [126,128]. However, Spinaci et al. found that treatment with low doses of green tea extract did not improve nuclear and cytoplasmic maturation of cumulus oocyte complexes (COCs) nor did it improve blastocyst formation in pigs [128].

Despite some controversial benefits regarding EGCG in assisted reproductive technology outcomes, studies have established a negative effect of the green tea extract on porcine granulosa cells. It inhibited cellular proliferation as well as steroidogenesis and angiogenesis. Levels of estrogen and progesterone dropped with EGCG (50 µM) as well as VEGF secretion [129]. In a study, progesterone release from rabbit ovarian fragments decreased and caspase-3 intracellular concentrations increased with EGCG (10, 100 µg/mL) treatment [130]. Hence, green tea might not be the best treatment option for infertility due to its anti-proliferative, pro-apoptotic, and anti-steroidogenic properties (Table 2 and Table 3).

Several clinical trials examined the effect of green tea on fertility. The FRIEND study, which is an ongoing randomized double-blind clinical trial, is currently determining the effect of EGCG on pregnancy rates in women with fibroids seeking fertility treatment with clomid-intrauterine insemination (IUI) (NCT05364008). Another double-blind, placebo-controlled study examined the effect of FertilityBlend, a nutritional supplement including green tea, chasteberry, and other vitamins and minerals, on progesterone levels, the menstrual cycle, basal body temperature, and pregnancy rates in women with infertility for three months. The investigators found an increase in progesterone levels, a normalization of the menstrual cycle, and an increase in pregnancy rates after treatment completion [131] (Table 4).

### 4.3. Green Tea and Uterine Fibroids

Uterine leiomyomas, also known as uterine fibroids, are the most common benign gynecological tumors. Fibroids affect up to 80% of reproductive-age women and cause a considerable healthcare burden [132,133]. Fibroids are composed of monoclonal cells that arise from the smooth muscle of the uterus, but the exact etiopathogenesis of their growth remains unclear [134]. Depending on their size and location, fibroids can present with bleeding symptoms (heavy, prolonged, or painful menses), bulk symptoms (constipation, urinary frequency, pelvic pressure), or both. Fibroids are also associated with subfertility [135]. Hormonal medications are commonly used to provide temporary symptom relief, but the definitive treatment remains surgical (myomectomy or hysterectomy) [136]. Both modifiable and non-modifiable risk factors have been associated with the development of fibroids. These include age, race, sex hormones, obesity, genetics, diet, and lifestyle factors. Several studies suggest that the consumption of certain nutrients, including green tea, affects fibroid growth [137,138].

**Figure 4:**
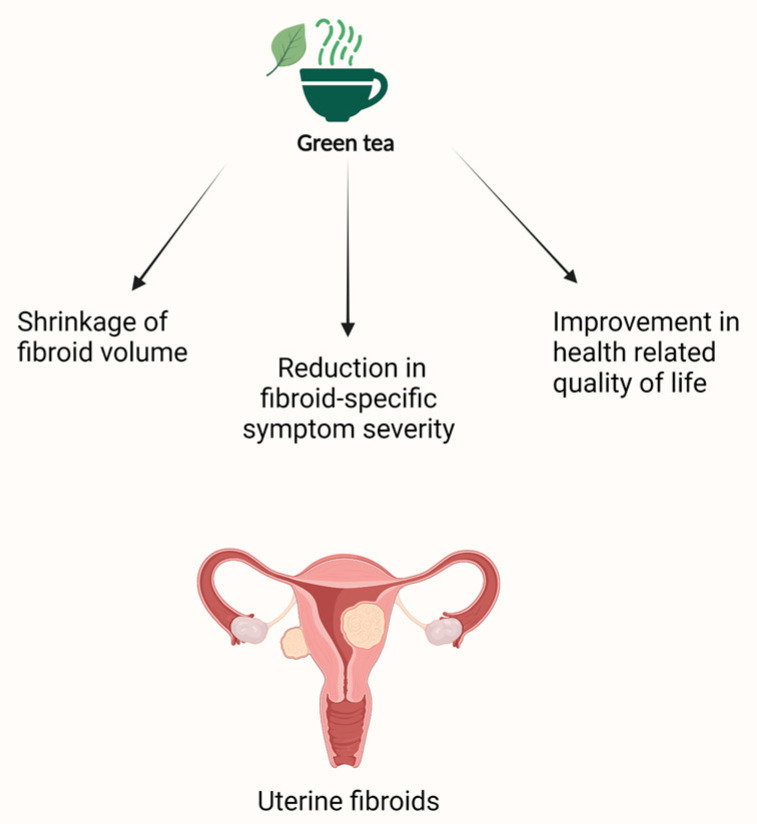
Schematic presentation of the effects of green tea on uterine fibroids.

Epigallocatechin-gallate (EGCG), the main antioxidant compound in green tea, has been widely studied for its antitumor properties in both gynecologic and non-gynecologic cancers [139,140]. The anti-proliferative and anti-angiogenic effects of EGCG on cancer cells provide insight into its potential protective role against benign tumors like fibroids. The inhibitory effect of EGCG on fibroid growth has been studied both in vitro and in vivo. Zhang et al. treated human leiomyoma cells with varying concentrations of EGCG and showed that EGCG inhibited cell growth and promoted apoptosis in a dose-dependent manner [141]. Similar effects were demonstrated in different animal models (Table 2 and Table 3). In one study, the consumption of 1.25 mg of EGCG per day for 4–8 weeks dramatically decreased fibroid size and weight in mice [142]. This was also observed in Japanese quail, where EGCG supplementation over a 12-month period reduced tumor volume and number [143]. In 2013, Roshdy et al. were the first to evaluate the effect of EGCG on symptomatic fibroids in humans. Thirty-nine women were randomized to take 800 mg of green tea extract or a placebo daily for 4 months. The EGCG-treated group had a 32.6% reduction in fibroid volume, as opposed to a 24.3% increase in fibroid volume in the placebo group. Moreover, EGCG was found to significantly improve symptom severity and quality of life in the treated group [144]. These findings are consistent with the results of two recent Italian studies in which EGCG was given in combination with vitamin B6 and vitamin D to a group of reproductive-age women over a period of 3 to 4 months. Both pilot studies reported significant shrinkage of fibroid size as well as improvements in patients’ quality of life in the treated group compared to the placebo group [145,146]. A similar combination of EGCG, vitamin D, and vitamin B6 was administered to 14 premenopausal women aged >40 years in a daily diary-based pilot study conducted by Grandi et al. [108]. Participants then reported fibroid-related symptoms (bleeding and pain) in a daily diary for 3 months. When compared to baseline, EGCG treatment was found to significantly decrease menstrual flow length in this group; however, no significant change in the health-related quality of life was observed (Figure 4; Table 4) [108].

Collectively, the aforementioned findings support the beneficial action of EGCG to reduce uterine fibroid growth, but the exact mechanism by which it exerts its inhibitory effect is yet to be determined. One suggested mechanism is the regulation of COMT enzyme activity and protein expression by EGCG. COMT gene polymorphisms have been associated with increased risk of uterine fibroids and are more common in African American women. Moreover, COMT has been shown to have much higher expression in leiomyomas compared to normal myometrium [147,148]. Aside from its role in catecholamine catabolism, COMT converts an antiestrogen compound (2-hydroxyestradiol) to a proestrogen (2-methoxyestradiol) and leads to an estrogen-rich environment. COMT is therefore thought to indirectly contribute to fibroid tumorigenesis, a highly estrogen-dependent process [149]. Interestingly, one study has demonstrated that EGCG inhibits COMT activity and protein expression in human leiomyoma cells in vitro [147]. Furthermore, treatment with EGCG modulated survival and stress signaling pathways in wild-type leiomyoma cells, but these effects were minimal or reversed in COMT-silenced leiomyoma cells. It is therefore suggested that the antigrowth effects of EGCG on fibroids might be in part mediated by COMT inhibition [147]. Another molecular target is bone morphogenic protein 2 (BMP2), an important regulator of cell differentiation and apoptosis. Endometrial resistance to BMP2 plays a crucial role in uterine fibroid pathogenesis and is thought to be provoked by the cytokine TGF-β. EGCG was found to upregulate BMP2 gene expression and decrease TGF-β, possibly leading to improved endometrial receptivity [147,150,151,152].

### 4.4. Green Tea and Endometriosis

Endometriosis is a common, chronic disease characterized by the ectopic implantation of endometrial-like (resembling the lining of the uterus) lesions in the pelvic area outside the uterine cavity. Although the precise cause of endometriosis is still a subject of research, theories such as retrograde menstruation and cellular metaplasia have been proposed [153]. The growth of endometriotic lesions is estrogen-dependent; accordingly, the condition predominantly affects women of reproductive age and is a frequent cause of pelvic pain and infertility [154]. The pain is typically cyclic as ectopic implants proliferate and shed during menses, but pain can also occur with intercourse (dyspareunia) and defecation (dyschezia), severely affecting a woman’s quality of life. Current treatment options are limited to analgesics, hormonal therapy (combined oral contraceptives, progestins, or gonadotropin-releasing hormone agonists), and surgery [155]. Even after surgical resection of the endometriotic lesions, there is a 50% chance of recurrence within five years of surgery [156].

**Figure 5:**
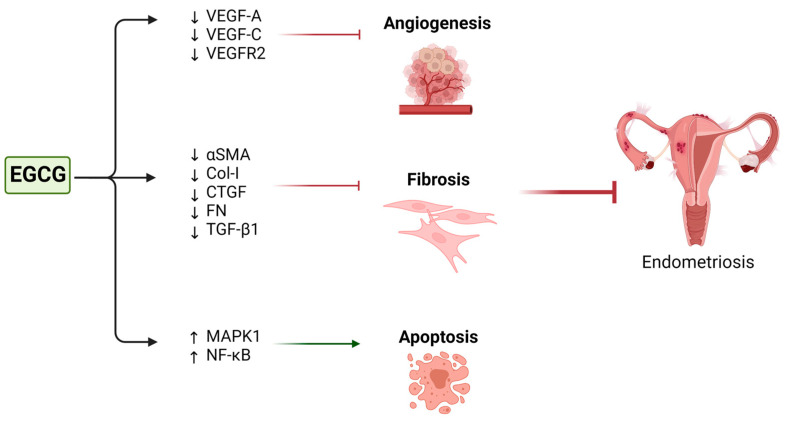
Schematic presentation of the possible roles and mechanisms of green tea in endometriosis. ↓ = downregulation; ↑ = upregulation.

A first mechanism that is thought to mediate EGCG’s protective role against endometriosis is apoptosis. The proapoptotic role of EGCG has been previously demonstrated in different cancer cells such as breast, pancreatic, hepatic, and prostate cancer cells, as well as ovarian and cervical cancer cells [94,95,157,158]. Although endometriosis is a benign disease, three studies have shown that EGCG treatment drove apoptosis in endometriosis cells [62,159,160] and increased the expression of apoptotic factors like NF-κB and mitogen-activated protein kinase 1 (MAPK1) (Figure 5; Table 2) **[159]**.

Numerous in vivo studies have been conducted using murine models of endometriosis to explore the role of EGCG. In each study, mouse endometrial tissues or, alternatively, human endometrium derived from patients with endometriosis were transplanted into mouse models to induce endometriosis. In these endometriosis models, mice were then treated with specific doses of EGCG (versus placebo) for 2–4 weeks [62,159,160,161,162,163,164]. Results consistently showed that EGCG treatment suppressed endometrial growth, neovascularization, and fibrosis [62,159,161,162,163,164], reduced the size and weight of endometriotic implants [62,159,160], and inhibited the development of new endometriotic lesions [163]. Comparable effects were observed in vitro, whereby EGCG inhibited the growth of human endometrial cell cultures derived from patients with endometriosis [160,162] and significantly suppressed estrogen-stimulated activation, proliferation, and pro-angiogenic factor expression in isolated hamster endometrial cells [163].

Many of these studies have also explored specific cellular signaling pathways targeted by EGCG in endometrial cells. First, EGCG was found to downregulate the expression of pro-angiogenic factors like vascular endothelial growth factor A (VEGF-A), vascular endothelial growth factor C (VEGF-C), and the tyrosine kinase receptor VEGF receptor 2 (VEGFR2) in murine endometriosis models [159,161]. Interestingly, EGCG did not affect the expression of hypoxia-inducible factor 1 (HIF1A), another important regulator of angiogenesis [159]. EGCG was shown to inhibit specific key factors of the VEGF/VEGFR2 angiogenesis signaling pathway like matrix metalloproteinase-9, c-JUN, interferon- g, and chemokines (C-X-C motif) [161]. It was therefore suggested that EGCG exerts its antiangiogenic effect on endometriosis mainly by interfering with VEGF signaling.

The antifibrotic effect of EGCG on endometriosis observed by Matsuzaki et al. [162] in mouse models was supported by their in vitro findings. EGCG treatment of endometrial and endometriotic stromal cells resulted in suppressed expression of fibrotic markers such as alpha-smooth muscle actin (α-SMA), type I collagen (Col-I), connective tissue growth factor (CTGF), and fibronectin (FN), and notably inhibited TGF-β1-mediated fibrotic signaling pathways (Table 3) [162].

The effect of EGCG on endometriosis has yet to be explored in humans. A randomized clinical trial in Hong Kong is currently recruiting patients with endometrioma to assess changes in endometriotic lesion size, growth, and neovascularization after 3-month treatment with 400 mg of EGCG twice daily versus placebo (NCT02832271) (Table 4).

**Table 2:** In vitro studies examining the effect of green tea on benign gynecologic disorders.

	Study and Year	Cell Lines	Green Tea Extract/EGCG Concentrations	Mechanism of Action	Effect
Fibroids	Zhang et al. (2010) [141]	HuLM	0, 0.1, 1.0, 10, 50, 100, and 200 µM	Upregulation of genes from the TGF-b and stress pathways, inhibition of the survival pathway and NFκB–dependent inflammatory pathway Decreased expression of PCNA, cdk4, and bcl2 Increased the expression BAX	Dose-dependent and time-dependent inhibition of cellular proliferation Increase in apoptosis
	Zhang et al. (2010) [142]	ELT3	200 μmol/L	Decreased expression of PCNA and Cdk4	Inhibition of cellular proliferation Induction of apoptosis
	Zhang et al. (2014) [147]	WT-HuLM and COMT-shRNA-HuLM	100 μM	Decreased COMT expression and enzyme activity Decreased expression of PCNA and Cdk4	Decreased proliferation of WT-HuLM cells but not COMT-shRNA-HuLM
Endometriosis	Laschke et al. (2008) [163]	Isolated hamster endometrial stromal and glandular cells	1. 40 µM EGCG. 2. 1 µM 17β-estradiol 3. 40 µM EGCG + 1 µM 17β-estradiol	Suppressed estrogen-stimulated activation, proliferation, and VEGF expression	Prevention of the development of new endometriotic lesions
	Xu et al. (2011) [161]	Human microvascular endothelial cells	10–50 µM	Suppressed VEGF-C expression and reduced VEGFR-2 and ERK activation	Inhibition of angiogenesis
	Ricci et al. (2013) [160]	Primary cultures of human endometrial epithelial cells from patients with endometriosis	0, 20, 40, 80, and 100 µM		Reduction in cell proliferation and increase in apoptosis
	Matsuzaki et al. (2014) [162]	Isolated endometrial and endometriotic stromal cells	50 or 100 µM	Decreased expression of the fibrotic markers αSMA, Col-I, CTGF, and FN Inhibition of TGF-β1-stimulated activation of MAPK and Smad signaling pathways	Inhibition of fibrosis in endometriosis
Infertility	Huang et al. (2021) [125]	Porcine cumulus oocyte complexes	5, 10, 20 µM	Reduction in MDA and ROS levels Increased GSH concentrations Increased expression of superoxide dismutase, catalase, and glutathione peroxidase Reduction of bax and caspase-3 expression Upregulation of bcl-2 expression Increase in blastocyst and cleavage rate	Increased oocyte maturation and antioxidant capacity
	Wang et al. (2007) [126]	Bovine cumulus oocyte complexes	15, 20 µM of green tea extract	Increased blastocyst formation Increased GSH levels	Increased oocyte maturation Improvements in embryonic development
	Barakat et al. (2014) [127]	Sheep cumulus oocyte complexes	0.3 mg/mL	Increased oocyte development to metaphase II Increased blastocyst formation	Increased nuclear maturation and embryo development
	Spinaci et al. (2008) [128]	Pig cumulus oocytes complexes	0 to 25 μg/ml	Decreased blastocyst formation at 25 μg/mL Inhibition of progesterone production	Inhibition of steroidogenesis
	Basini et al. (2005) [129]	Swine granulosa cells	5, 50 μg/ml	Decreased E2 and P4 production Decreased VEGF Increased H_2_O_2_ and superoxide dismutase activity	Inhibition of cellular proliferation, steroidogenesis, and angiogenesis
	Balasi et al. (2019) [130]	Rabbit ovarian fragments	0, 1, 10, 100 μg/ml	Upregulation of caspase-3 Inhibition of P4 and testosterone	Inhibition of steroidogenesis Increase in apoptosis

**Table 3:** In vivo studies examining the effect of green tea on benign gynecologic disorders.

	Study and Year	Animal Model	Green Tea Extract/EGCG Concentrations	Mechanism of Action	Effect	Side Effects
Fibroids	Zhang et al. (2010) [142]	Female athymic nude mice	1.25-mg EGCG/mouse/day for 4 and 8 weeks	Decreased expression of PCNA and Cdk4	Reduction of fibroid tumor size and weight	None reported
	Ozercan et al. (2008) [143]	Japanese quail (Coturnix coturnix japonica)	200 or 400 mg EGCG/kg for 12 months	Decreased serum and liver malondialdehyde and TNF-α concentrations	Decreased incidence and size of leiomyomas	None reported
Endometriosis	Laschke et al. (2008) [163]	Syrian golden hamster dorsal skinfold chamber model	65 mg/kg EGCG for 14 days	Decreased microvessel number and density Decreased centerline red blood cell velocity and volumetric blood flow	Reduced angiogenesis and blood perfusion Induced regression of endometriotic lesions	None reported
	Xu et al. (2011) [161]	SCID mice	50 mg/kg/day EGCG for 3 weeks	Suppressed VEGFC/VEGFR2 expression and signaling pathway through c-JUN, interferon-γ, matrix metalloproteinase 9, and chemokine (C-X-C motif) ligand 3 pathway	Inhibition of angiogenesis	None reported
	Guan et al. (2020) [164]	BALB/c female nude mice	8.333 mg/mL EGCG for 16 days	Increased E-cadherin expression Reduced DNA methylation of the E-cadherin promoter region	Inhibition of endometrial lesion growth	None reported
	Ricci et al. (2013) [160]	BALB/c mouse model	1.20 or 100 mg/kg EGCG for 4 weeks	Decreased cell proliferation, reduced vascular density, and increased apoptosis within the lesions	Inhibition of the development and reduction in the size of endometriotic lesions	None reported
	Matsuzaki et al. (2014) [162]	Female nude mice	50 mg/kg/day EGCG for 21 or 8 days	Decreased scores for Sirius Red and Masson trichrome staining in EGCG-treated mice. Lower staining score for human CD10-positive cells in untreated mice than in treated mice	Prevention of fibrosis progression in endometriosis	None reported
	Xu et al. (2009) [159]	SCID mice	50 mg/kg/day EGCG for 2 weeks	Down-regulation of VEGF-A expression Up-regulation of Nuclear factor-kappa B and mitogen-activated protein kinase 1 expression Reduction in mean total number and size of endometrial microvessels	Inhibition of angiogenesis Increase in apoptosis	None reported
	Wang et al. (2013) [62]	NOD-SCID mice transplanted with endometrial tissues from CMV-Luc mic	50 mg/kg pro-EGCG (EGCG octaacetate). or 50 mg/kg EGCG for 4 weeks	Increased total apoptotic cell numbers in the lesions Decreased microvessel parameters Decrease in both the CD31-positively and αSMA-negatively stained new microvessel numbers and the CD31-positively and αSMA-positively stained old microvessel numbers in the lesion	Inhibition of growth and angiogenesis Increase in apoptosis	None reported
Adenomyosis	Chen et al. (2013) [165]	Mice treated with 1 mg/kg tamoxifen to induce adenomyosis	5 or 50 mg/kg EGCG for 3 weeks	Decreased plasma corticosterone levels Reduced uterine contractility and suppressed myometrial infiltration	Improvement in adenomyosis- induced pain, stress, and uterine hyperactivity	None reported
	Chen et al. (2014) [166]	Mice treated with 1 mg/kg tamoxifen to induce adenomyosis	5 or 50 mg/kg EGCG for 3 weeks	Increase in GABAergic inhibition manifested by an increase in glutamate decarboxylase (GAD) 65-expressing neurons in the brainstem nucleus raphe magnus (NRM) Decreased expression of p-p65, cyclooxygenase 2, oxytocin receptor, collagen I and IV, and transient receptor potential vanilloid type 1 in ectopic endometrium or myometrium Reduced number of macrophages infiltrating into the ectopic endometrium Increased expression of progesterone receptor isoform B(PR-B)	Attenuated stress response and improved hyperalgesia through the GABAergic system in the brain Reduced myometrial infiltration	None reported
Infertility	Balazi et al. (2019) [130]	Nulliparous rabbit	Food enriched with 5 g and 20 g of green tea for 302 days	Increased ovarian length Decreased conception and kindling rates Decreased number of liveborn and weaned pups	Inhibition of steroidogenesis Increase in apoptosis	Decrease in platelet distribution width
PCOS	Ghafurniyan et al. (2015) [167]	PCOS rats	50, 100, 200 mg/kg of hydro-alcoholic extract containing 200 g of dried green tea leaves powder for 10 days		Decreased weight and insulin resistance Decreased testosterone level	None reported

**Table 4:** Clinical trials examining the effect of green tea on benign gynecologic disorders.

	Study	Study Design	Intervention vs. Control	Outcomes	Treatment Duration	Results	Side Effects
Fibroids	Roshdy et al. [144]	Randomized, double-blinded clinical trial N = 33 22 treated and 11 control	Intervention: 800 mg of green tea extract daily Control: placebo (800 mg of brown rice) daily	Primary outcome: total fibroid volume Secondary outcome: mean change in fibroid specific symptom severity and health-related quality of life (UFS-QOL)	4 months	32.6% reduction in fibroid volume in the group treated with green tea extract vs. 24.3% increase in the placebo group. 32.4% reduction in fibroid-specific symptom severity and significant improvement (18.53%) in health-related quality of life in the treatment group compared to the placebo group.	None reported
	Grandi et al. [108]	Pilot prospective daily-dairy based study N = 16	A combination of EGCG (300 mg), Vitamin B6 (10 mg), and VD (50 mg) daily No control	Primary outcome: reduction of uterine fibroid (UF) size Secondary outcomes: - Change of health-related quality of life (QoL) and sexual life - Change in bleeding profile and pelvic pain per day of bleeding	3 months	17.8% reduction in UF’s median size compared to baseline. No significant changes in health-related quality of life or sexual life after treatment. Significant decrease in menstrual flow length (0.9 day). No change in cycle length, menstrual flow intensity, or menstrual pain intensity.	None reported
	Porcaro et al. [145]	Pilot trial N = 30 15 treated and 15 control	Intervention: one tablet of 25 μg vitamin D + 150 mg EGCG + 5 mg vitamin B6 twice daily Control: no treatment	Primary outcome: change of fibroid volume Secondary outcomes: variation of the number of fibroids, distress caused by heavy menstrual bleeding, pelvic pressure, fatigue, quality of life (QoL), and the severity of symptoms (SS)	4 months	34.7% decrease in fibroid volume in the treated group vs. 6.9% increase in the control group. Improvement in the QoL of women in the treatment group vs. a slight decrease in QoL in the control group. Reduction of the SS in the treatment group vs. no variation in the control group.	None reported
	Miriello et al. [146]	N = 95 41 treated and 54 control	Intervention: one tablet of 25 μg vitamin D + 150 mg EGCG + 5 mg vitamin B6 twice daily Control: no treatment	Primary outcome: change of fibroid volume Secondary outcomes: variation of myomas color score, distress by heavy menstrual bleeding, pelvic pain, and quality of life	4 months	37.9% decrease in fibroid total volume in the treated group vs. 5.5% increase in the control group. Decrease in the peripherical fibroid vascularization (color score 2) in 7.7% of treated patients vs. 5.5% increase in color score 2 in the control group. Significant improvement in pelvic pain in the treated group vs. no change in the control group. The decrease in heavy bleeding in the treated group compared to the control group was not statistically significant. Significant improvement in the quality of life of treated women compared to their pre-treatment baseline.	None reported
Infertility	NCT05364008	Pilot trial N = 200	Intervention: 1650 mg low caffeine green tea extract Control: 1650 mg placebo matching smell, taste, color, and texture	Primary outcome: cumulative live birth rate Secondary outcomes: conception rate, miscarriage rate, change of fibroid volume, change of fibroid symptom severity score, change of health-related quality-of-life questionnaire score, and time of conception	6 months	Ongoing trial	None reported
	Westphal et al. [131]	Double blind placebo-controlled study N = 93 53 were treated and 40 control	Intervention: FertilityBlend Control: placebo	Outcomes: changes in progesterone levels, basal body temperature, length of menstrual cycle, pregnancy rate, and incidence of side effects	3 months	Increase in mean mid-luteal progesterone, basal body temperature, and normalization of the menstrual cycle in the treated group compared to the control group. 14 of the 53 women in the intervention group were pregnant. No significant side effects.	None reported
Menopause	Rondanelli et al. [168]	Double-blind placebo-controlled randomized trial N = 28 14 were treated and 14 control	Intervention: 150 mg Greenselect Phytosome Control: placebo matching size, shape, color, odor, and taste	Primary outcomes: respiratory quotient (RQ), percentages of carbohydrates (% CHO) and fat oxidation (%FAT), and resting energy expenditure (REE) Secondary outcomes: body composition, glucose and lipid profiles, inflammatory state, liver and kidney functions, and hormonal and catecholamines status	60 days	Statistically significant decrease in RQ, in % CHO and fat oxidation, in waist circumference, in insulin, as well as in inflammatory markers in the treated group compared to the control group. Statistically significant increase in adiponectin, noradrenalin, MB, % LIP, and the adiponectin/leptin ratio in the treated group compared to the control group.	None reported
PCOS	Tehrani et al. [169]	Double-blind randomized trial N = 60 30 were treated and 30 control	Intervention:500 mg green tea tablet Control: placebo	Outcomes: free testosterone, fasting insulin levels, and weight	12 weeks	Decrease in free testosterone as well as insulin levels and weight in the treated group compared to the control group.	Gastrointestinal side effects
	Mombaini et al. [170]	Randomized double-blind placebo-controlled clinical trial N = 50 25 were treated and 25 control	Intervention: 500 mg green tea tablet Control: placebo corn starch tablets	Outcomes: anthropometric indices and inflammatory markers	45 days	Significant decrease in BMI, waist circumference, and body fat percentage in the treated group compared to the control group. No significant difference in inflammatory markers (IL-6, CRP, and TNF- α) between the treated and control groups.	Gastrointestinal side effects
	Chan et al. [171]	Randomized placebo-controlled clinical trial N = 34 18 were treated and 16 control	Intervention: capsules containing 2% freeze-dried tea powder Control: placebo	Outcomes: anthropometric measurements, biochemical and hormonal profiles	3 months	No statistically significant difference in BMI, body weight, and body fat, fasting insulin, glucose, cholesterol, HDL, LDL, testosterone, SHBG, free androgen, androstenedione, DHEA-S, LH, and FSH between the intervention and control groups.	No significant side effects

### 4.5. Green Tea and Adenomyosis

Adenomyosis is a benign gynecologic disorder characterized by the infiltration of the uterine myometrium by endometrial glands and stroma [172]. The disorder commonly affects women in their later childbearing years, between the ages of 35 and 50. The etiology is not well understood, and many clinical symptoms overlap with those of other benign entities like endometriosis and fibroids. Patients can be asymptomatic or present with dysmenorrhea, heavy menstrual bleeding, and chronic pelvic pain. A diffusely enlarged, “boggy” uterus on examination is characteristic [173,174]. Some evidence also suggests that adenomyosis can interfere with tubal transport and endometrial function, causing infertility [175].

Only a few studies in the literature have explored the relationship between green tea and adenomyosis. In one study, mice were treated with 2 mg/kg tamoxifen to induce adenomyosis, then randomly assigned to receive low-dose EGCG (5 mg/kg), high-dose EGCG (50 mg/kg), or no treatment for 3 weeks. Outcomes measured included plasma corticosterone level (an indicator of stress), depth of myometrial infiltration, and uterine contractility. The investigators observed that adenomyosis induction in mice caused an increase in plasma corticosterone levels, possibly attributed to adenomyosis-induced stress and hyperalgesia [165]. EGCG treatment was shown to decrease plasma corticosterone levels, suppress myometrial infiltration, and reduce uterine contractility in mice induced with adenomyosis [165] (Table 3).

In a follow-up study, the same group observed that mice induced to have adenomyosis had a remarkable loss of inhibitory gamma-aminobutyric acid (GABA)-ergic neurons in the brainstem nucleus raphe magnus (NRM), indicative of hyperalgesia. Treatment with EGCG increased the number of these neurons and was suggested to attenuate the hyperalgesia associated with adenomyosis in these models [166]. Together, these findings provide insight into the promising effects of EGCG against adenomyosis, but further research on this topic is clearly warranted.

### 4.6. Green Tea and PCOS

PCOS is one of the most common causes of infertility among women. It affects approximately 6–12% of women in the United States [176]. It is an endocrine disorder characterized by a hyperandrogenic state and insulin resistance. Green tea components, especially catechins, have the potential to provide health benefits to women with PCOS.

An in vivo study examining the effect of green tea on PCOS-induced rats showed that intraperitoneal injection of hydro-alcoholic green tea extract (50, 100, and 200 mg/kg) reduced the rats’ weight and improved insulin resistance and ovarian morphology [167]. No change in fasting insulin levels was noted, but a significant decrease in HOMA-calculated insulin resistance and fasting glucose was found. Green tea also decreased testosterone levels in PCOS-induced rats [167] (Table 3).

**Figure 6:**
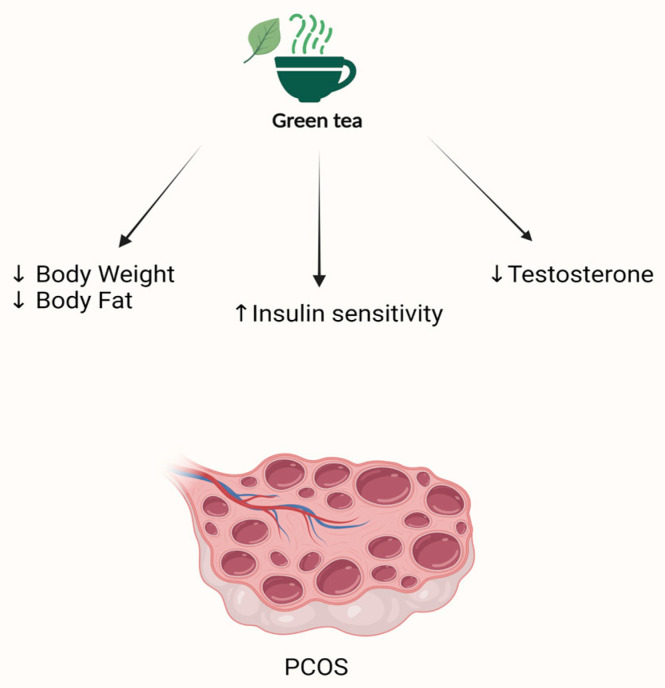
Schematic presentation of possible benefits of green tea in PCOS. ↓ = downregulation; ↑ = upregulation.

Three clinical trials examined the effects of green tea capsules on women with PCOS. Regarding weight loss and insulin sensitivity, one study found that overweight and obese women who had a daily dose of green tea tablets showed a significant drop in body weight as well as an improvement in insulin sensitivity compared to the placebo group [169]. However, two other trials did not show a significant reduction in body weight, but one of them did observe a decrease in body fat [170,171]. Chen et al. found no difference in both fasting insulin and glucose in obese women with PCOS after a three-month treatment with capsules, including a 2% freeze-dried tea powder [171]. Regarding the hyperandrogenic state, while a study found that green tea decreased testosterone levels in women with PCOS [169], Chen et al. did not detect a difference [171]. No change was observed in SHBG, testosterone, androstenedione, free androgen, DHEA-S, LH, or FSH in either the intervention or placebo groups [171]. The pathogenesis of PCOS may involve chronic inflammation. Despite its anti-inflammatory properties, there was no change in inflammatory markers in women with PCOS who consumed green tea [170,171] (Table 4; Figure 6).

### 4.7. Green Tea and Menopause

Menopause—is diagnosed after 12 months of the last period. Menopausal symptoms can be very bothersome for women and range from hot flashes, mood changes, weight gain, and osteoporosis—all related to the depletion of estrogen levels.

Regular green tea consumption in postmenopausal women decreased body fat, especially visceral fat, as well as the lipid profile, which reduced the women’s risk of cardiovascular diseases. Total cholesterol and LDL levels were reduced compared to non-drinkers. HDL remained the same between both groups [177]. EGCG also positively affected body composition in postmenopausal women. A double-blind clinical trial sought to evaluate the effect of 60 days of supplementation with green tea extract on adipose tissue dysfunction. In overweight and obese postmenopausal women, a reduction in percent body fat oxidation, in waist circumference, in insulin, as well as in inflammatory markers, was observed in the intervention group [168] (Table 4).

In addition to the benefits of green tea on the cardiovascular system, this compound also plays a role in improving bone health in postmenopausal women. Menopausal women are at increased risk for osteoporosis because of their estrogen deficiency, which accelerates bone resorption. Shen et al. reported that supplementation of oophorectomized 15-month-old rats with green tea extract (0.1%, 0.5%) resulted in an increase in bone mineral density and bone mineral content [178]. EGCG (1, 10 µmol/L) can improve the osteogenic differentiation of murine bone marrow mesenchymal stem cells and mitigate bone loss by increasing expression of alkaline phosphatase as well as osteogenesis-related genes, eventually enhancing mineralization [179]. De novo bone formation was also stimulated by an EGCG-induced increase in BMP2 [179]. EGCG (50–100 µmol/L) also reduced osteoclastogenesis by nuclear translocation and NF-κB transcription in RAW 264.7 cells [180]. It also mitigated bone loss by decreasing Sema4D expression in bone tissue [181].

Postmenopausal women who regularly consumed green tea had a strengthened bone metabolism and a reduced risk of fracture. Several studies showed that women over the age of 60 who consumed a daily dose of green tea had higher bone mineral density than those who did not [182]. In addition, a daily dose of green tea before the onset of menopause was preventive against osteoporosis. It was associated with a higher bone mineral density in the postmenopausal age compared to women who did not drink green tea regularly [183]. Consequently, green tea helped prevent osteoporosis by reducing oxidative stress, enhancing osteoblastogenesis, and reducing osteoclastogenesis [184].

Furthermore, a study conducted on postmenopausal women found lower levels of estrogen in those who drank green tea regularly compared to non- or irregular green tea drinkers. Plasma estrone and estradiol, as well as androstenedione, were lower in regular green tea drinkers. This finding suggests that green tea might be beneficial in reducing the risk of breast cancer in post-menopausal women, which can be driven by a hyperestrogenic state [185].

## 5. EGCG: Safety and Side Effects

Individuals in any culture worldwide consume large amounts of green tea daily. Each 240-mL serving of green tea contains approximately 187 mg of EGCG, with many individuals consuming upwards of three cups a day [186]. In addition, there are many supplements on the market ranging from 25 mg to upwards of 750 mg of green tea catechins per serving [186]. Due to this wide range in consumption and supplemental availability, the dosage of green tea used in clinical settings varies widely across studies, ranging from 50 mg to over 1600 mg [187]. It has been suggested that a safe consumption level is 338 mg/day for solid doses and 704 mg/day for green tea beverage consumption [186], though higher levels have been used for clinical intervention studies without adverse side effects [188].

EGCG was generally well tolerated without side effects in many studies, although side effects have been reported [186,189]. In many studies discussed in this manuscript, women reported no adverse effects [108,131,144,145,146,169]. Potential side effects include headache, fatigue, dizziness, diarrhea, indigestion, nausea, constipation, and, most seriously, liver inflammation [189]. Many of these side effects were found to be dose- or drug administration-dependent, with GI events correlating with high doses (400–4000 mg/day) and nausea occurring most frequently in studies with capsule administration [186]. Many of the safety concerns surrounding the use of EGCG and other green tea compounds in clinical settings relate to the impact they could have on liver function and inflammation. In 2020, the United States Pharmacopeia (USP) Green Tea Extract Hepatotoxicity Expert Panel published a comprehensive review of clinical data (from January 1966 and June 2007) on green tea extracts (GTE) concerning liver damage [190] and raised the issue of the potential for GTE to cause hepatotoxicity [190]. Reported instances of acute liver injury are common at very high dosages (above the current recommended thresholds) [191]. However, even at high dosages, these events remain rare, with fewer than 100 total reported instances as of 2020 [192]. For example, in a recent study of the hepatic safety of EGCG in the treatment of uterine fibroids, 720 mg green tea extract tablets were shown to have no impact on liver function [188]. In rare cases of acute liver injury, symptoms and effects are largely mitigated upon stopping treatment [192,193,194]. Additionally, in instances of hepatotoxicity, capsule administration was more significantly correlated with heightened liver enzymes in comparison to beverage consumption [186]. This was also true generally, as green tea in beverage form was more tolerable than a solid bolus dosage and was thus correlated with fewer instances of side effects [189]. Overall, green tea and its major catechin, EGCG, have been shown to be both safe and tolerable, with side effects primarily affecting patients treated at exceedingly high dosages.

## 6. Conclusions

To conclude, green tea, with its major bioactive component EGCG, shows promise for health benefits in the treatment of benign gynecologic disorders. In vitro studies indicated that EGCG may act on several intracellular signaling pathways involved in the pathogenesis of uterine fibroids and endometriosis. Animal studies further supported the role of EGCG in the shrinkage of fibroid and endometriotic lesions. In humans, EGCG was found to alleviate symptoms associated with fibroids, PCOS (weight gain, insulin resistance, and hyperandrogenism), and menopausal sequelae (osteoporosis and weight gain). The role of green tea in the treatment of infertility remains controversial, and more studies are needed to draw definitive conclusions about its effect on adenomyosis. As most evidence is derived from in vivo and in vitro experimental studies, clinical studies that evaluate the effects of EGCG on the development and growth of benign gynecological diseases should be considered.

## Figures and Tables

**Table 1:** Green tea nutritional information panel (source: U.S. DEPARTMENT OF AGRICULTURE).

Nutrition Facts	
Serving size (g)	100
Energy (kcal)	1
Total Carbs (g)	0
Protein (g)	0.22
Fats (g)	0
Vitamin B1 (thiamine) (mg)	0.007
Vitamin B2 (riboflavin) (mg)	0.058
Vitamin B3 (niacin) (mg)	0.03
Vitamin B6 (mg)	0.005
Vitamin C (mg)	0.3
Iron (mg)	0.02
Potassium (mg)	8
Sodium (mg)	1
Magnesium (mg)	1

## Data Availability

The data presented in this study are available in the article and tables.
